# Ant Queen Egg-Marking Signals: Matching Deceptive Laboratory Simplicity with Natural Complexity

**DOI:** 10.1371/journal.pone.0004718

**Published:** 2009-03-05

**Authors:** Jelle S. van Zweden, Jürgen Heinze, Jacobus J. Boomsma, Patrizia d'Ettorre

**Affiliations:** 1 Centre for Social Evolution, Department of Biology, University of Copenhagen, Copenhagen, Denmark; 2 Biology I, University of Regensburg, Regensburg, Germany; University of Exeter, United Kingdom

## Abstract

**Background:**

Experiments under controlled laboratory conditions can produce decisive evidence for testing biological hypotheses, provided they are representative of the more complex natural conditions. However, whether this requirement is fulfilled is seldom tested explicitly. Here we provide a lab/field comparison to investigate the identity of an egg-marking signal of ant queens. Our study was based on ant workers resolving conflict over male production by destroying each other's eggs, but leaving queen eggs unharmed. For this, the workers need a proximate cue to discriminate between the two egg types. Earlier correlative evidence indicated that, in the ant *Pachycondyla inversa*, the hydrocarbon 3,11-dimethylheptacosane (3,11-diMeC_27_) is more abundant on the surface of queen-laid eggs.

**Methodology:**

We first tested the hypothesis that 3,11-diMeC_27_ functions as a queen egg-marking pheromone using laboratory-maintained colonies. We treated worker-laid eggs with synthetic 3,11-diMeC_27_ and found that they were significantly more accepted than sham-treated worker-laid eggs. However, we repeated the experiment with freshly collected field colonies and observed no effect of treating worker-laid eggs with 3,11-diMeC_27_, showing that this compound by itself is not the natural queen egg-marking pheromone. We subsequently investigated the overall differences of entire chemical profiles of eggs, and found that queen-laid eggs in field colonies are more distinct from worker-laid eggs than in lab colonies, have more variation in profiles, and have an excess of longer-chain hydrocarbons.

**Conclusions:**

Our results suggest that queen egg-marking signals are significantly affected by transfer to the laboratory, and that this change is possibly connected to reduced queen fertility as predicted by honest signaling theory. This change is reflected in the worker egg policing response under field and laboratory conditions.

## Introduction

Conflict over male production in insect societies is often resolved by worker policing [1]–[3], where workers either destroy each other's eggs or show aggressive behavior towards reproductive workers to discourage their egg laying [4]–[7]. Since policing workers preferentially destroy worker-laid eggs (WLE) relative to queen-laid eggs (QLE), they need an accurate cue or set of cues to decide which eggs to eliminate, e.g. an honest queen egg-marking pheromone that workers are unable to mimic [8], [9]. It has been hypothesized that this cue may reflect the fertility of queens, since fertility is the major parameter that differs between queens and workers in social insects. Moreover, workers may rely on information regarding the reproductive state of the queen for regulating their own reproduction [9], [10]. For the honeybee, *Apis mellifera*, it has been suggested that the Dufour's gland secretion of the queen, and possibly only the ester fraction of this secretion, plays a role in egg marking [11]. Honeybee QLE and WLE also have distinct hydrocarbon profiles, but these seem to be of little importance for worker policing [12]. In spite of clear theoretical predictions and an extensive search for the honeybee egg-marking pheromone, no conclusive evidence of the identity of this signal, and of its honesty as a signal of reproductive quality, has been obtained [13], [14].

**Figure 1:**
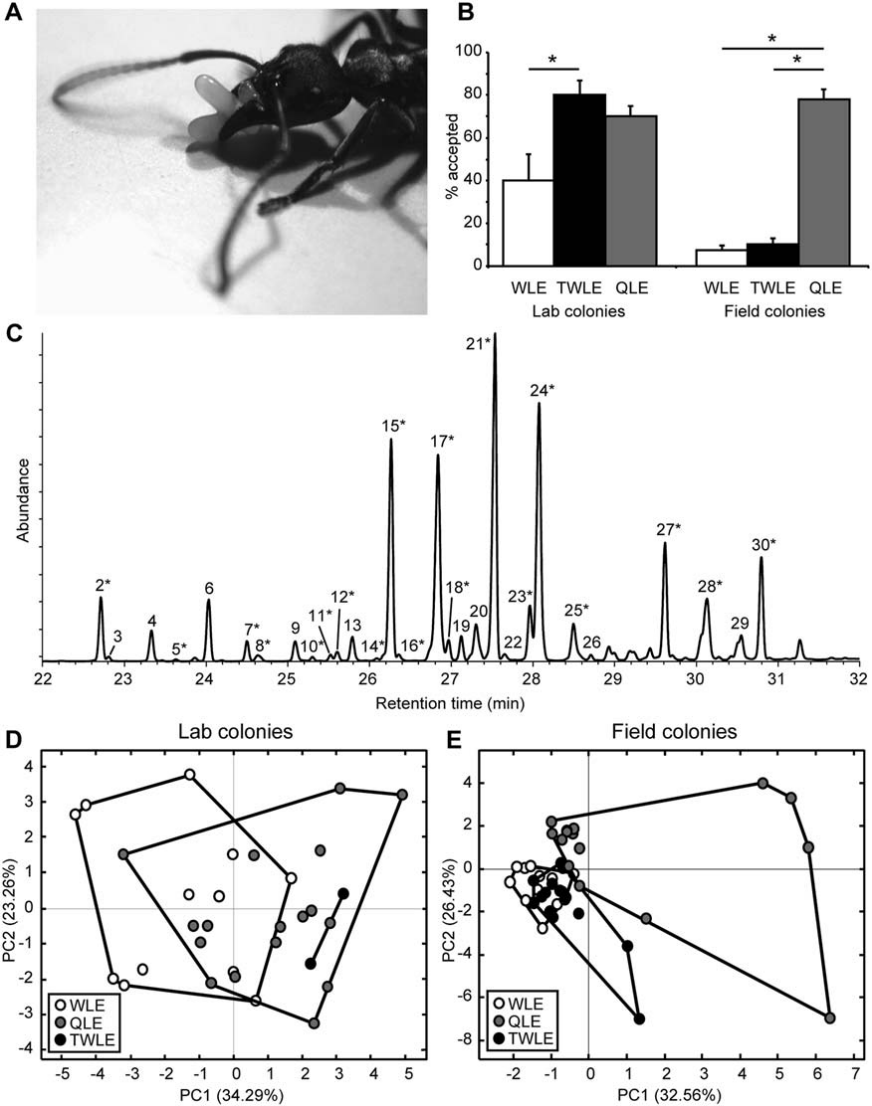
The effect of 3,11-diMeC_27_ on the acceptance of eggs in discriminator colonies and on the overall chemical profile. (A) A worker of *Pachycondyla inversa* carrying eggs. (Photo by M.A. Fürst and J.S. van Zweden). (B) The average acceptance rates in discriminator colonies (±S.E.) of sham-treated worker-laid eggs (WLE), worker-laid eggs treated with 3,11-diMeC_27_ (TWLE), and sham-treated queen-laid eggs (QLE) in lab and field colonies; * indicates p<0.05 (GLZ, see text). (C) A typical hydrocarbon profile of a *Pachycondyla inversa* egg: the 19 compounds marked with an asterisk were used in PCA models (see Methods); labels and compounds correspond to those in a previous study [17]: 2 = C_25_, 3 = unknown, 4 = 11-,13-MeC_25,_ 5 = 5-MeC_25_, 6 = 3-MeC_25_, 7 = C_26_, 8 = 3,9-diMeC_25_, 9 = 10-,11-,12-MeC_26_, 10 = 6-MeC_26_, 11 = 4-MeC_26_, 12 = 2-MeC_26_, 13 = 3-MeC_26_, 14 = unknown, 15 = C_27_, 16 = 2,11-diMeC_26_, 17 = 9-,11-,13-MeC_27_, 18 = 7-MeC_27_, 19 = 5-MeC_27_, 20 = 9,13-diMeC_27_, 21 = 3-MeC_27_, 22 = 5,9-diMeC_27_, 23 = C_28_, 24 = 3,11-diMeC_27_, 25 = 10-,11-,12-,13-,14-MeC_28_, 26 = 6-MeC_28_, 27 = C_29_, 28 = 11-,13-,15-MeC_29_, 29 = 11,15-diMeC_29_, 30 = 3-MeC_29_. (D,E) Plots of the first two principal components showing the chemical similarity of the QLE and WLE and the predicted position of the TWLE.

The ant *Pachycondyla inversa* (Figure 1A) is one of the few other social insects for which significant effort has been put in identifying the queen egg-marking pheromone. Queens – and egg-laying workers in queenless colonies – typically have a higher abundance of the hydrocarbon 3,11-dimethylheptacosane (3,11-diMeC_27_) on their cuticle than non-laying workers. Across queens, the abundance of 3,11-diMeC_27_ is associated with ovarian development [15], [16] and the surface of queen-laid eggs is known to contain more of this compound than worker eggs [17], [18]. Moreover, in lab colonies 3,11-diMeC_27_ is preferentially detected by the antennae of workers from an entire spectrum of compounds on the egg surface [16], consistent with its inferred pheromonal activity. However, explicit experimental tests to establish whether 3,11-diMeC_27_ is indeed the queen egg-marking pheromone have not been done.

In the present study, we provide a series of tests of the role of 3,11-diMeC_27_ for the acceptance of QLE versus WLE by other workers of *P. inversa*. We applied synthetic 3,11-diMeC_27_ [synthetic pathway described in ref. 16] on WLE of laboratory-maintained colonies (hereafter “lab colonies”) and found that this hydrocarbon, as predicted from the above-mentioned correlative evidence, increases the acceptance rate of WLE to the same level as QLE. However, when repeating the same experiments with freshly collected field colonies (hereafter “field colonies”) on a plantation in Brazil there was no effect of treating WLE with 3,11-diMeC_27_, which prompted us to do a full scale chemical study of the egg surface hydrocarbon blends expressed under both laboratory and field conditions.

## Results

Queen-laid eggs (QLE), worker-laid eggs (WLE), and 3,11-diMeC_27_-treated worker-laid eggs (TWLE) were introduced into discriminator colonies, and their fate (accepted or policed) was observed. In lab colonies, TWLE were significantly better accepted than sham-treated WLE (Figure 1B; Generalized Linear Model, WLE vs. TWLE, χ^2^ = 5.38, df = 1, p<0.05), and their survival was not significantly different from QLE (TWLE vs. QLE, χ^2^ = 0.46, df = 1, p = 0.50), suggesting that 3,11-diMeC_27_ does indeed allow workers to discriminate between QLE and WLE. However, this result could not be confirmed when the same policing assays were repeated with field colonies (Figure 1B): TWLE were not better accepted than WLE (WLE vs. TWLE, χ^2^ = 0.32, df = 1, p = 0.57), and both were significantly less accepted than QLE (WLE vs. QLE, χ^2^ = 56.61, df = 1, p<0.0001, TWLE vs. QLE, χ^2^ = 46.77, df = 1, p<0.0001).

If 3,11-diMeC_27_ by itself was a universal queen egg-marking pheromone for *P. inversa*, the same increase in acceptance rate should have been achieved in both field and lab colonies, regardless of the similarity of the rest of the hydrocarbon profile of eggs. However, if the acceptance rate of discriminating workers also depended on other egg compounds, or even on the entire chemical profile of eggs, this might provide an explanation for the observed difference, provided that there would be systematic differences in chemical profiles of eggs between field and lab colonies. The hydrocarbon profiles of both QLE and WLE consist of linear and methyl-branched alkanes, the majority of which could be identified [29 compounds; Figure 1C; see also [ref. 17]]. Additional samples of eggs, relative to those used in bio-assays, showed that in lab colonies treatment increased the average (±SD) percentage of 3,11-diMeC_27_ in the entire hydrocarbon profile from 9.0% (±2.7%) on WLE (n = 12) to 26.1% (±0.6%) on TWLE (n = 2), as compared to 12.0% (±3.2%) on QLE (n = 17). Similarly, in field colonies the relative abundance increased from 6.4% (±2.3%) on WLE (n = 14) to 23.5% (±8.2%) on TWLE (n = 14), compared to 12.9% (±7.8%) on QLE (n = 15) (see also Figure S1). Hence, QLE and WLE had similar relative proportions of 3,11-diMeC_27_ under lab and field conditions, and treatment with synthetic 3,11-diMeC_27_ had a clear and comparable effect on the relative abundance of this compound in the profile. We used log-transformed peak areas in Principal Component Analysis (PCA), and when plotting the first two PC's for QLE and WLE of lab colonies, we found considerable overlap between the two egg types (Figure 1D) with the predicted positions of TWLE falling within the QLE group. Hence, the major difference between QLE and WLE in lab colonies is indeed the relative abundance of 3,11-diMeC_27_, so that the experimental addition of this compound to WLE turns them into mimics of QLE. In the field colonies, however, there was a much clearer distinction between QLE and WLE to start with (Figure 1E), and the PCA positions of TWLE did not come to overlap with those of QLE. This implies that, although treatment with 3,11-diMeC_27_ worked both under field and laboratory conditions, the effect in field colonies was not sufficient to let TWLE mimic QLE, but the same chemical compound made TWLE as acceptable as QLE in lab colonies.

**Figure 2:**
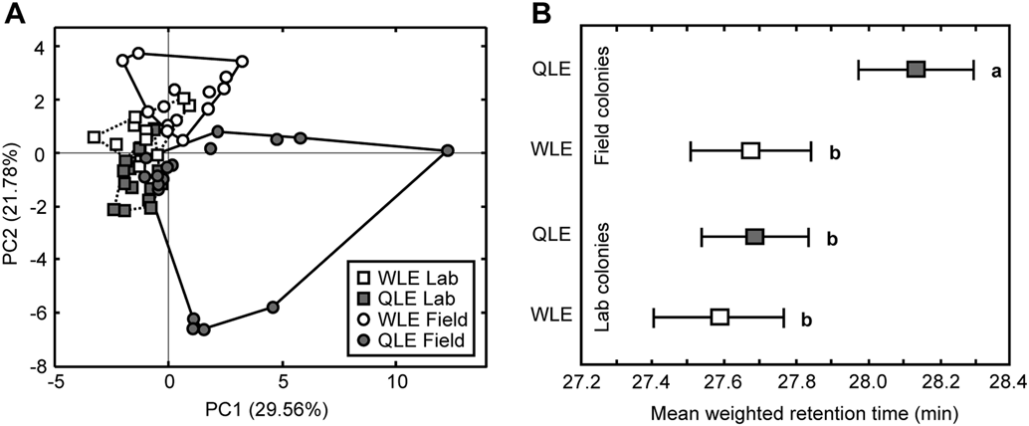
The overall egg surface hydrocarbon analyses. (A) A plot of the first two principal components showing the overall chemical similarity of queen-laid eggs (QLE) and worker-laid eggs (WLE) in both lab and field colonies. (B) The mean weighted retention times (±95% c.l.) of QLE and WLE in field and lab colonies. Different letters indicate significant differences between groups (p<0.01, ANOVA with post-hoc comparisons of least square means, see text).

Apart from more variation and better separation between QLE and WLE in field colonies (Figure 2A), the profiles of QLE in field colonies were characterized by higher abundance of longer-chain hydrocarbons (3-MeC_27_, C_28_, 3,11-diMeC_27_, C_29_, and especially 3-MeC_29_), which was only observed to some extent on QLE in lab colonies [15], [17]; see also Text S1 and Figure S2. Formal analysis based on the mean weighted retention times of all detected compounds confirmed that the QLE of field colonies had significantly longer hydrocarbons on their surface than field-produced WLE or either of the egg types in lab colonies (Figure 2B; ANOVA, F_3,54_ = 9.08, p<0.0001). 3,11-diMeC_27_ is a relatively long-chained hydrocarbon (retention time of 28.1 min with our temperature program), so that its experimentally increased abundance must have increased the average chain length of all compounds on the surface of WLE to such extent that they came to overlap with QLE in lab colonies, but not in the field colonies where the difference between QLE and WLE was much larger (Figure 2B).

## Discussion

We have tested whether 3,11-diMeC_27_ acts as the queen-marking pheromone of the ant *Pachycondyla inversa* in the context of worker policing. The experimental addition of this single compound to the surface of WLE was sufficient to convey a reliable signal under lab conditions, but in field colonies it no longer elicited the predictable effect on egg acceptance. This shows that the compound in itself cannot be the universal signal that *P. inversa* workers use to distinguish between QLE and WLE. The discrepancy between lab and field behavioral results was supported by the finding that, when considering entire hydrocarbon profiles, TWLE mimicked QLE under lab conditions, but not under field conditions. In field colonies, we found that QLE on average have significantly heavier surface hydrocarbons than WLE, whereas this was not so in lab colonies, which might indicate that the queen egg marking signal is primarily encoded in a longer average chain length of cuticular hydrocarbons. We hypothesize that the excess of heavy hydrocarbons may be connected to a higher fertility of field queens compared to lab queens and that these compounds are costly to produce and/or maintain [19], so that queen quality is honestly signaled by the average chain length, rather than the abundance of a single compound.

### Uniformity of cuticular profiles under laboratory conditions

A general consequence of imposing laboratory conditions is that replicas are as uniform as possible. This has many advantages for controlled experiments, but for ants and other social insects receiving the same food may result in chemical profiles becoming more similar across unrelated colonies [20]. Indeed, our lab colonies not only showed a greater overlap between QLE and WLE, but also less variation in egg hydrocarbon profiles across colonies (Figure 2A). This may have been a consequence of all individuals receiving the same food, but may also have been affected by the fact that in the field workers generally go out foraging, and thus are exposed to different temperature, humidity and light conditions than queens. In the lab, on the other hand, all individuals live under the same physical conditions.

Another field of biology where the contrast between field variability and lab uniformity is well known, is that of quantitative genetics. Artificial selection experiments typically overestimate the heritability of traits relative to natural populations, and a central problem is therefore how these differences can be matched [e.g. 21], [22], [23]. Similarly, conditions during laboratory rearing of fruit flies have been shown to affect mating [24] and feeding behavior [25], just as uniform laboratory conditions affect nestmate recognition in social insects. Studies on ants [26]–[28], wasps [29] and honeybees [30] have all shown that prolonged maintenance under uniform conditions can diminish the discrimination efficiency between nestmates and non-nestmates, quite possibly because of converging hydrocarbon profiles [20]. Deceptive laboratory results have been obtained in studies of chemical recognition in vertebrates. Individual recognition in genetically uniform laboratory strains of the house mouse, *Mus musculus domesticus*, has traditionally been assumed to depend on scents derived from the major histocompatibility complex (MHC). However, this finding has recently been challenged by a study on wild strains with larger genetic variability, where major urinary proteins (MUPs) appeared to be more important [31]. Examples like these emphasize that great care should be taken when performing bioassays and interpreting results under laboratory conditions, as they are not always representative for the more complex field conditions.

### Possible fertility reduction under laboratory conditions

The pattern we observed in our experiments may also be due to another feature of our laboratory conditions, namely a possible reduction in fertility of queens. Fertility signaling is of major importance in social insects, as workers sacrifice their own reproduction for inclusive fitness benefits via the reproduction of their fertile queen. The queen signal has been hypothesized to reflect fertility [8], [9], and is thus expected to vary according to the reproductive state of the queen. In the laboratory, we tried to reproduce the natural conditions of *P. inversa* in terms of temperature, humidity and photoperiod, but conditions may well have remained sub-optimal, because colony productivity never approached the natural situation (e.g. lab colonies produced an average±SD of 3.0±4.7 new workers in the 35 day study period, whereas field colonies produced 16.6±5.4 new workers). The move to the lab may thus have reduced queen fecundity, which could also have induced the higher acceptance of sham-treated WLE in lab colonies relative to field conditions (40.0% vs. 7.5%, respectively; Figure 1B). In field colonies, we assume that queens were highly fecund, and we observed that they produced eggs that were chemically very different from WLE, so that the workers could easily recognize and destroy WLE in field colonies. However, if fecundity dropped under laboratory conditions, the queen signal may have decreased so that her eggs became chemically more worker-like (although the difference in 3,11-diMeC_27_ remained) and workers failed more easily to discriminate between WLE and QLE (and an increase 3,11-diMeC_27_ may have tipped the balance towards acceptance). Alternatively, the workers could have lowered their threshold for WLE acceptance after detecting the decrease in queen fecundity, indicating that she was failing or ageing. In any case, this would imply that workers police eggs by relative comparison to known eggs [see also ref. 32], which allows them to react in a condition-dependent manner.

### The hypothesis of queen signal encoded in the average chain length of cuticular profiles

Strong differences in multiple hydrocarbons between fertile and unfertile individuals have been found in several social insect species. Similar to our results, in the ant *Harpegnathos saltator*, egg-layers are characterized by the presence of 13,23-diMeC_37_ (the very longest hydrocarbon in this species), but also by a generally higher average chain length of their hydrocarbon profiles [33]. Egg-layers of another Ponerine ant, *Gnamptogenys striatula*, characteristically have six additional methyl-branched alkanes on the heavy end of the spectrum [34]. Similarly, the fertility signal of *Streblognathus peetersi* appears to be encoded in a higher relative abundance of some of the heaviest alkenes (C_35:1_ to C_39:1_) [35]. Queens of *Formica fusca* exhibit a set of alkanes and alkenes with a chain length higher than 29 carbon atoms [36], which are not found on the cuticles of workers [37]. Also, QLE of *Vespula vulgaris* have a higher abundance of alkenes of 25, 27 and 29 carbon atoms and a longer average chain-length than WLE (J.S. van Zweden and P. d'Ettorre, unpublished results).

On the other hand, in the species *Camponotus floridanus* [6], *Linepithema humile* [38] and *Aphaenogaster cockerelli* [39] queen fertility is associated with large qualitative shifts towards shorter average chain length of hydrocarbons. Also in *Myrmecia gulosa* [40] the chemical profile of fertile individuals is characterized by two compounds, 9-C_25:1_ and 3-MeC_25_, both of which are short compared to the rest of the profile. In *Diacamma ceylonense,* fertile females can be significantly discriminated from sterile females on the basis of four methylated alkanes, two of which are among those with the shortest chain length in this species [41]. In *Dinoponera quadriceps*, however, the compounds characterizing the alpha female, 9-C_31:1_ and 9-C_33:1_, are found among the median length hydrocarbons of the profile [42]. Similarly, in *Lasius niger* queens are characterized by a higher abundance of C_31:1_, C_33:1_, and especially 3-MeC_31_, which are spread throughout the profile (L. Holman, S. Dreier and P. d'Ettorre, in preparation). These varying results imply that considerable further work is needed to establish whether these caste-correlated differences would decrease when queen fecundity is compromised.

Although these studies mainly obtained correlative results, they suggest that these putative fertility signals are making use of the production of alkenes and shifts in the average hydrocarbon chain length, which should therefore be more costly to produce/maintain than alkanes of median chain length [19]. To concentrate on the chain length, as may be the case for *P. inversa*, this hypothesis predicts that cuticular hydrocarbon profiles of unfertile workers of social insects should be bell-shaped, with abundant peaks in the centre (e.g. 27 carbon atoms in the case of *P. inversa*) and minor peaks at both ends. This is indeed what is generally observed across the social insect species investigated so far [Figure 1C; e.g. [refs. 6], 36, 40]. Chain length of hydrocarbons is regulated by fatty acyl-CoA elongase reactions, and the position of the double bond in alkenes is determined by a fatty acyl-CoA desaturase before the elongation process starts [43]. Not enough is known about the synthetic pathway of the different hydrocarbons to accurately estimate their cost, but any switch or addition in the metabolic pathways will likely entail a cost, so that honesty might (partly) be maintained by this mechanism. In any case, the multi-component nature of the hydrocarbon signals seems to be crucial, which would allow the receiver to make an accurate quality assessment of the signaler [44], [45] and the signal to remain corruption-proof. This is both because a greater number of metabolic pathways increases the overall cost of signal production and because the overproduction of a single component by cheaters may be easy to ignore or may even solicit aggression towards the signaler [c.f. 9,46]. Hence, the time may have come to revise the “single pheromone – single message” model, just as we have earlier given up on the “single gene – single behavior” paradigm.

## Methods

### Study organisms

Colonies of *Pachycondyla inversa* (Formicidae: Ponerinae) [47] were collected in November of two consecutive years from a cocoa plantation near Ilhéus, Bahia, Brazil. In 2004, four polygynous (2–3 queens, 38±17 workers (mean±SD)) and four queenless colonies (24±7 workers) were brought to Regensburg, Germany (“lab colonies”). Colonies were kept under near-natural conditions (27°C and 60% humidity, 12L:12D photoperiod) and in plastic boxes (19×19×9 cm) with a regularly moistened plaster floor. A chamber in the plaster (6×6×1.5 cm) served as nest cavity. Diluted honey and cockroaches, *Nauphoeta cinerea*, were provided three times per week, and water was provided *ad libitum*. Experiments started five weeks after establishment in the lab, when the first WLE appeared in queenless colonies.

In 2005, five polygynous (2–4 queens, 40±9 workers), five monogynous (1 queen, 26±7 workers) and seven queenless colonies (19±5 workers) were collected and kept in plastic boxes (26×15×8 cm ) with a moist plaster floor under natural conditions for humidity, temperature and day-night cycle, i.e. in a building on the plantation itself (“field colonies”). These are the same colonies as used by van Zweden *et al*. [7], to test for specialization in policing behavior. Two chambers (2.8×2.8×0.9 cm) in the plaster served as nest cavities. Diluted honey, termites and crickets were provided three times per week, and water was provided *ad libitum*. Experiments started a few days after the first WLE appeared in queenless colonies, i.e. approximately two weeks after collection.

### Policing assays

To mimic queen-laid eggs (QLE), we treated worker-laid eggs (WLE) with synthetic 3,11-diMeC_27_ (see [16] for pathway of synthesis). First, 20 µl of pentane containing 0.1 µg µl^−1^ 3,11-diMeC_27_ was dispensed onto a clean glass cover slip (24×24 mm). After the pentane had evaporated, only the compound remained on the cover slip. An egg was carefully removed from its source colony (0–48 hours after oviposition), placed onto the cover slip, and rolled back and forth for 3 min in the area where the compound was present, so that the concentration on the surface of the egg increased (“treated worker-laid eggs” or TWLE, hereafter). Sham-control WLE and QLE were treated in a similar way, but using pentane without any 3,11-diMeC_27_. After treatment, the eggs were individually introduced into a discriminator colony and their acceptance or rejection [cf. 7,17] was assessed during a 30 min observation period immediately following introduction (Figure 1B).

The four polygynous lab colonies were used as discriminator colonies in egg policing trials, following a standard protocol [17]. Discriminator colonies were paired with egg-supply colonies, so that the QLE (n = 10 per discriminator colony) were always obtained from only one of the other polygynous colonies, and the WLE (n = 10) and TWLE (n = 10) from one of the queenless colonies. The five polygynous field colonies were used as discriminator colonies, whereas the monogynous field colonies provided the QLE (n = 10) and the queenless field colonies the WLE (n = 16) and TWLE (n = 12). In this case, each discriminator colony received QLE and WLE from all source colonies in equal quantities. In all assays only hetero-colonial eggs were introduced, just as in earlier studies [7], [17]. Introduced TWLE and WLE always came from the same source colony. Statistical analyses were done using SAS 9.1.

### Chemical analysis

Surface chemicals of additional QLE, WLE, and TWLE (all after treatment on a cover slip) were extracted by individually washing the eggs in 30 µl of HPLC-grade pentane for 1 min. We injected 2 µl of this extract into an Agilent Technologies 6890N gas chromatograph, either equipped with a flame ionization detector (FID) and an Rtx-5 30.0 m×0.25 mm×0.50 µm capillary column (lab colonies), or with an Agilent 5975 mass selective detector (MS) and a HP-5MS column (30.0 m×0.25 mm×0.25 µm) (field colonies). For samples run with FID, compounds had been identified beforehand using GC-MS. The injectors were of the *split-splitless* type and the carrying gas was Helium at 1 ml min^−1^. The temperature program for the analysis of eggs from lab colonies ranged from 70°C to 180°C at 20°C min^−1^, then increased to 280°C at 5°C min^−1^, and was held at 280°C for 15 min. For the field colonies the temperature rose from 70°C to 180°C at 20°C min^−1^, then increased to 320°C at 5°C min^−1^ at which it was held for 5 min. The two temperature programs gave the same results in terms of detected compounds, which were identical to the compounds found by d'Ettorre *et al*. [17], except for compound 1 (an unknown non-hydrocarbon) that could not be found again. For each profile the peak area of each of twenty-nine peaks (Figure 1C) was integrated. For the lab colonies, we used WLE (n = 12) coming from four queenless source colonies and QLE (n = 17) from four queenright source colonies. TWLE (n = 2) came from two queenless source colonies. For the field colonies, WLE (n = 14) and TWLE (n = 14) from seven queenless source colonies were used, whereas QLE (n = 15) came from five monogynous source colonies.

For use in principle component analysis (PCA), the peak areas were normalized using a Z-transformation, to reduce the “closure” effect [i.e. the relative contribution of one peak affecting the relative contribution of the rest of the compounds; ref. 48]. We included only those peaks that varied more between QLE and WLE than within either of these groups, to decrease noise in the analysis, i.e. for which the standard deviation over all QLE and WLE samples combined was at least as large as the pooled standard deviation within these groups [49]. This reduced the number of variables to the nineteen peaks (Figure 1C – marked with an asterisk), out of twenty-nine that were available. A PCA including all variables gave similar results, so that this restriction was not essential. We performed PCA in LATENTIX 1.00 for Windows (MathWorks Inc., Denmark) to estimate the chemical similarity of the different egg profiles. This program allows validation of the PCA model with a test set, i.e. a set of samples that is not included to calculate the principle components, but plotted on the model afterwards by using the principle components. This procedure allowed us to keep the TWLE out of the initial analysis of total variable space, so that their position compared to QLE and WLE could be unambiguously predicted, because they had not influenced the model.

To investigate the relative intensity of short-chain *vs.* long-chain hydrocarbons, the mean weighted retention time was calculated by multiplying the average retention time of each peak with the relative area of that peak and taking the sum of these values over the entire profile. The mean weighted retention times were calculated for individual QLE and WLE of both field and lab colonies, and were subsequently used in ANOVA in STATISTICA 7.1.

## Supporting Information

Figure S1 Chromatograms of a typical queen-laid egg (QLE), a worker laid egg (WLE) and a worker-laid egg treated with 3,11-diMeC27 (TWLE). The treatment was successful in increasing the relative abundance of 3,11-diMeC27 on the surface of the eggs. Arrows point to 3,11-diMeC27.
(0.10 MB TIF)

Figure S2 Plots of the first two principal components showing the chemical similarity of QLE and WLE, using only three relatively long-chained linear and two 3-methyl alkanes as variables, in A) lab colonies and B) field colonies. The positions of compounds (PC loadings) are indicated with black dots. The QLE of field source colony P3 are marked separately, because these eggs grouped with WLE and their policing rate was similar to that of WLE (6 out of 10 eggs policed).
(0.17 MB TIF)

Text S1 Chain-length hypothesis and policing rates in individual colonies
(0.04 MB DOC)
