# Supplementary figures and images for: Ant Queen Egg-Marking Signals: Matching Deceptive Laboratory Simplicity with Natural Complexity
Source: PLoS One. 2009 Mar 5;4(3):e4718. doi: 10.1371/journal.pone.0004718 (PMC2648039; doi:10.1371/journal.pone.0004718)

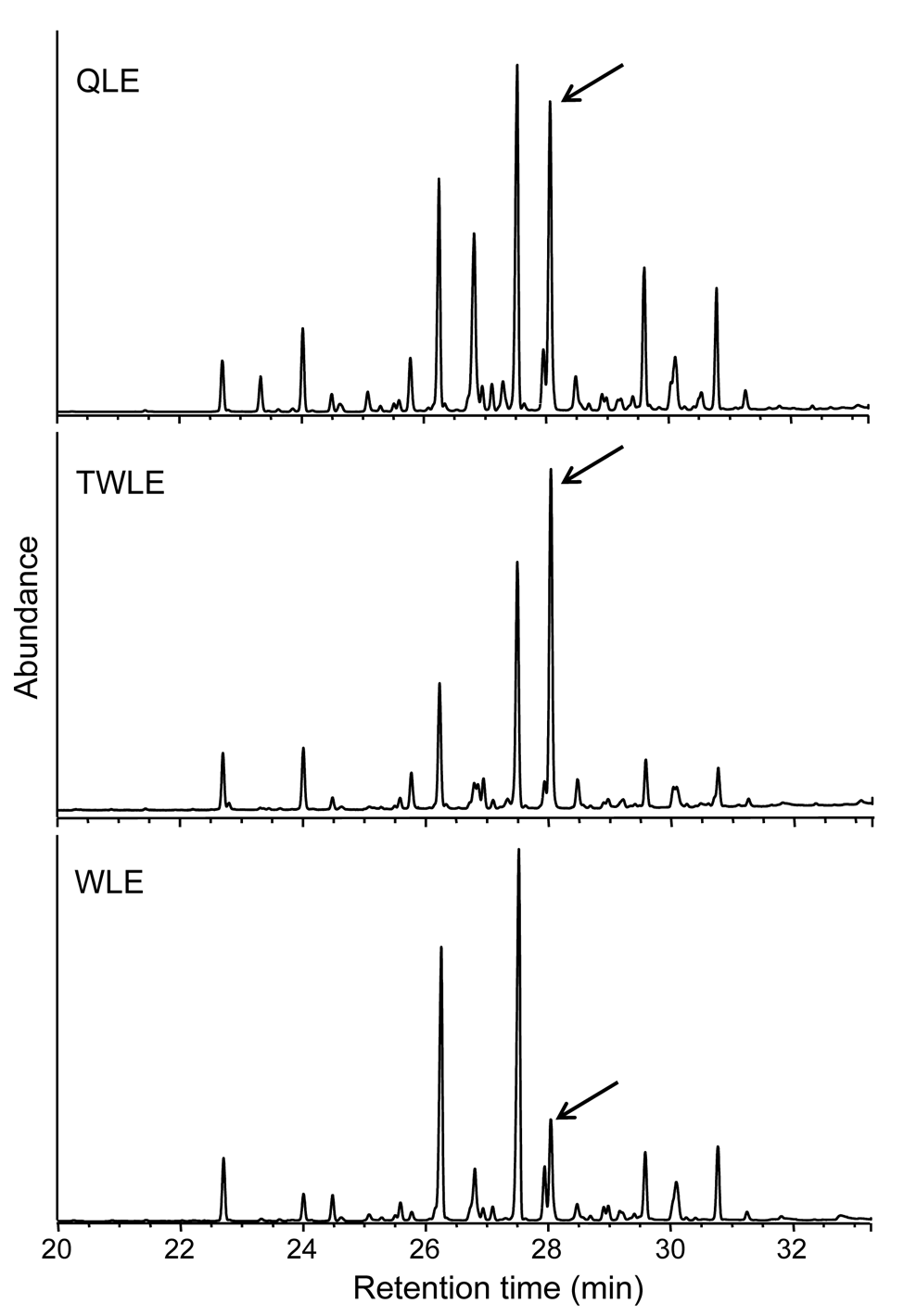

Supplement: Figure S1 — Chromatograms of a typical queen-laid egg (QLE), a worker laid egg (WLE) and a worker-laid egg treated with 3,11-diMeC27 (TWLE). The treatment was successful in increasing the relative abundance of 3,11-diMeC27 on the surface of the eggs. Arrows point to 3,11-diMeC27. (0.10 MB TIF) [file pone.0004718.s001.tif]

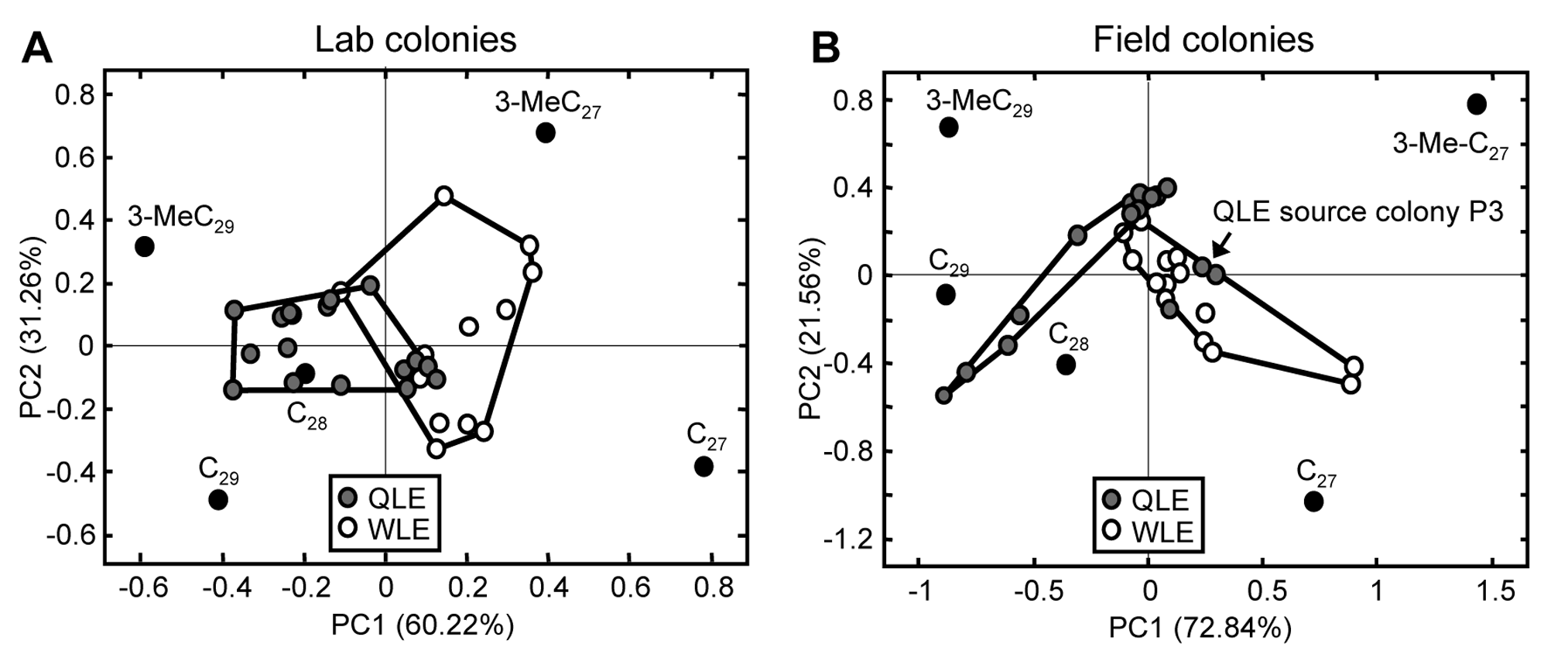

Supplement: Figure S2 — Plots of the first two principal components showing the chemical similarity of QLE and WLE, using only three relatively long-chained linear and two 3-methyl alkanes as variables, in A) lab colonies and B) field colonies. The positions of compounds (PC loadings) are indicated with black dots. The QLE of field source colony P3 are marked separately, because these eggs grouped with WLE and their policing rate was similar to that of WLE (6 out of 10 eggs policed). (0.17 MB TIF) [file pone.0004718.s002.tif]
