# Supplementary material for: Ant Queen Egg-Marking Signals: Matching Deceptive Laboratory Simplicity with Natural Complexity
Source: PLoS One. 2009 Mar 5;4(3):e4718. doi: 10.1371/journal.pone.0004718 (PMC2648039; doi:10.1371/journal.pone.0004718)
Supplement: Text S1 — Chain-length hypothesis and policing rates in individual colonies (0.04 MB DOC) [file pone.0004718.s003.doc]

### Chain-length hypothesis and policing rates in individual colonies

Profiles of QLE across field colonies were highly variable: on average QLE had many compounds in quantities >1% (C_25_, 3-MeC_25_, C26, 3-MeC_26_, C_27_, 9-, 11-, 13-MeC_27_, 7-MeC_27_, 5-MeC_27_, 9,13-diMeC_27_, 3-MeC_27_, C_28_, 3,11-diMeC_27_, 10-, 11-, 12-, 13-, 14-MeC_28_, C_29_, 11-, 13-, 15-MeC_29_, and 11,15-diMeC_29_, 3-MeC_29_), but eggs from two field colonies showed only five compounds (C_27_, 3-MeC_27_, C_28_, C_29_, 3-MeC_29_) in quantities >1%, and had only traces of 3,11-diMeC_27_. The latter eggs, however, were not policed at any higher rate than QLE from other field colonies (0/10 and 2/10 eggs policed, compared to 2.2/10 eggs policed on average). The five compounds that were on these eggs were all relatively long-chained compared to the average hydrocarbon spectrum of QLE (eluded after 26.2min in our temperature program, see also Figure S1), which supports the hypothesis of chain length playing a role in queen-marking. An alternative hypothesis that the queen signal is encoded in only these five compounds only would not work, as that would fail to explain our results with TWLE in lab colonies (see results section and figure 1B of the main text).

To illustrate that the major overall difference between QLE and WLE lies in the average chain length, rather than in the relative abundance of 3,11-diMeC_27_ alone, we performed PCA with only these five compounds that all eggs have in common. We found QLE and WLE to be well separated under both field and laboratory conditions, with longer hydrocarbons characterizing QLE (Figure S2A, S2B). Moreover, we found that eggs collected from a single source colony of QLE with relatively shorter hydrocarbons grouped together with WLE and indeed experienced a significantly higher rate of policing relative to eggs from the other QLE source colonies from the field (Colony P3: 6/10 eggs policed, average: 2.2/10 eggs policed; χ^2^ = 14.45, df = 4, p < 0.01).
